# A Survey of Insulin-Dependent Diabetes—Part II: Control Methods

**DOI:** 10.1155/2008/739385

**Published:** 2008-06-12

**Authors:** Daisuke Takahashi, Yang Xiao, Fei Hu

**Affiliations:** ^1^Department of Computer Science, The University of Alabama, Tuscaloosa, AL 35487, USA; ^2^Computer Engineering Department, Rochester Institute of Technology, Rochester, NY 14623, USA

## Abstract

We survey blood glucose control schemes for insulin-dependent diabetes therapies and systems. These schemes largely rely on mathematical models of the insulin-glucose relations, and these models are typically derived in an empirical or fundamental way. In an empirical way, the experimental insulin inputs and resulting blood-glucose outputs are used to generate a mathematical model, which includes a couple of equations approximating a very complex system. On the other hand, the insulin-glucose relation is also explained from the well-known facts of other biological mechanisms. Since these mechanisms are more or less related with each other, a mathematical model of the insulin-glucose system can be derived from these surrounding relations. This kind of method of the mathematical model derivation is called a fundamental method. Along with several mathematical models, researchers develop autonomous systems whether they involve medical devices or not to compensate metabolic disorders and these autonomous systems employ their own control methods. Basically, in insulin-dependent diabetes therapies, control methods are classified into three categories: open-loop, closed-loop, and partially closed-loop controls. The main difference among these methods is how much the systems are open to the outside people.

## 1. INTRODUCTION

Complexity of a human biological system typically allows its relations to be expressed only in a nonlinear way. Because of this complexity, it is not simple to achieve insulin-dependent diabetic therapies autonomously. Diabetes mellitus is a metabolic disorder of endogenous insulin allowing excessive amount of glucose to stay in blood. In general, blood glucose is transformed into energy required by human activities, such as, walking, and this transformation requires insulin functionality. However, in diabetes mellitus, since a human body fully or partially lacks the insulin functionality, unchanged glucose remains in blood. A condition of high blood glucose profiles results in several complications, such as, eye, kidney, and nerve damage, called hyperglycemia [1]. Thus, in order to avoid the hyperglycemia, a continuous supply of exogenous insulin is required, and the insulin-dependent diabetic therapy usually does this. On the contrary, too much insulin supply may lead to a condition of low blood glucose profiles resulting in drowsiness, mental malfunctioning, irritability, and loss of consciousness [1]. This condition is called hypoglycemia and also dangerous to the diabetic. Thus, the insulin-dependent diabetic therapy must concern both hyperglycemia and hypoglycemia by providing an appropriate amount of exogenous insulin timely.

At the beginning of the insulin-dependent diabetic therapy, it is required to obtain an approximation of the insulin-glucose relation. This relation is usually described in a number of mathematical equations. Two methods are taken in this process, namely, empirical and fundamental methods. Arguably, this process is most time consuming.

Based on mathematical equations representing the insulin-glucose mechanism, therapies are systematically established. Broadly, controlling the blood glucose levels is achieved by means of three strategies, namely, open-loop, closed-loop, and partially closed-loop schemes. In general, the fully and partially closed-loop schemes involves several medical devices but the open-loop scheme does not. While in the closed-loop scheme, a system is aimed to completely encompass the diabetic, open- and partially closed-loop require the physician's contribution to complete the loops. Therefore, typically any decisions of the insulin injections are made by a physician in open- and partially closed-loop schemes. We explain these three strategies along with applications in the later sections.

This paper is the second part of our survey of insulin-dependent diabetes. Our first paper [2], the first part of the survey, mostly spent its pages on the background of insulin-dependent diabetes therapy, such as, description of type 1 and type 2 diabetes, the insulin functionality, and medical devices involved in the insulin-dependent therapy. In this paper, we survey blood glucose control schemes which lie on the basics of the insulin-dependent diabetes therapies and systems.

The rest of the paper is organized as follows. In Section 2, we briefly summarize diabetes mellitus for the sake of induction to the topic. In Section 3, we explain empirical and fundamental schemes to derive mathematical models of the insulin-glucose dynamics. From Sections 4 through 6, we explore the control strategies, especially for the insulin-glucose dynamics. In these sections, we provide several applications based on the controls. Finally, we conclude this survey in Section 7.

## 2. TYPE 1 AND TYPE 2 DIABETES

The World Health Organization (WHO) reported that there were currently nearly 180 million patients suffering from diabetes allover the world, and the number of the diabetics would increase more than 350 million people by 2030 [3]. From the same report, approximately 1.1 million people died from diabetes in 2005 and among this number, people under 70 years old account for a half [3]. In the United States, currently it costs 136 billion dollars annually to take care of 12 million diabetes patients [4, 5]. In general, diabetes is considered as a condition that disproportionately affects developed countries.

Diabetes first emerged around 2000 B.C. while insulin and its functionality were discovered in 1921. Since the discovery of insulin, insulin-dependent diabetes therapies mostly concern how to delay the emergence of the complications in use of insulin supplement [6, 7]. In short, diabetes is characterized in a condition that blood keeps high glucose levels unchanged into energy resulting in several complications. Although insulin is largely concerned with this reaction, diabetes fully or partially lacks this functionality [8]. Diabetes eventually causes cardiovascular disease, chronic renal failure, retinal damage, nerve damage, and microvascular damage.

Besides, according to characteristics of diabetes, it is typically classified into two types, namely, type 1 and type 2 diabetes [8]. In short, in type 1 diabetes, from the malfunction of the pancreas resulting from the destruction of the *β* cells of the Islets of Langerhans, a supply of endogenous insulin completely stops. This requires other sources of insulin supplementation. Otherwise, the diabetic eventually falls into a condition of hyperglycemia. On the other hand, in type 2 diabetes, the insulin functionality gradually weakens, but does not completely stops. Since the diabetic more or less has the endogenous insulin supply, diabetes therapies mostly focus on exercises or regimens consuming or suppressing excessive glucose in blood. However, both type 1 diabetes and type 2 diabetes are considered chronic and currently incurable.

As mentioned before, type 1 diabetes completely stops the insulin supply. This is caused by the malfunction of the pancreas destroying the *β* cells which are responsible for the endogenous insulin supply. It is considered a reason why the destruction of the *β* cells occurs is due to the immune system which should react an infection by viruses, such as, the Coxsackie virus family or German measles but mistakenly destroys the *β* cells [9]. Type 1 diabetes is sometimes called childhood, juvenile or insulin-dependent diabetes although it does not only emerge during a childhood [9].

On the other hand, type 2 diabetes does not stop the endogenous insulin supply, but instead it is characterized in insulin resistance, insulin deficiency, and hyperglycemia [10]. Although in type 2 diabetes, endogenous insulin still can facilitate its functionality, it is largely degraded and cannot sufficiently change blood glucose into energy [11]. Thus, the amount of unchanged blood glucose will get larger resulting in hyperglycemia, a condition of high blood glucose profiles, causing eye, kidney, and nerve damage [12]. However, since in the early stage of type 2 diabetes, the symptoms are not serious or noticeable, it is likely to miss its emergence easily. This causes the diabetes more serious and critical. Type 2 diabetes is sometimes inherited genetically, but in most of the cases it caused from irregular life styles, such as, the lack of exercise, obesity or a sedentary lifestyle [10]. Type 2 diabetes is also called non-insulin-dependent, obesity-related, or adult-onset diabetes.

Currently, diabetes can be treated at home by a patient himself or herself under the supervision of a physician. During the earlier years of the diabetes treatment, logs and tables of insulin injections and regimens were kept, and according to these records next insulin injections and regimens as well as exercises are determined by a physician. Now, microcontrollers and sensors enable autonomous insulin-dependent diabetes therapies systematically adjusting the insulin supply. More precisely, according to feedback from one or more blood glucose sensors, a rate of insulin supply of an insulin pump is determined, which works like an “artificial pancreas.” The advantages of an “artificial pancreas” are safe, automatic, and nonintrusive. Several control schemes are developed in order to achieve the optimal exogenous insulin supply suppressing the blood glucose levels within a safe range of nominal. For more details about diabetes fundamentals, please refer our first paper in [2].

## 3. MODELING THE HUMAN INSULIN-GLUCOSE SYSTEM

To procure the mathematical models of the human insulin-glucose system, several approaches are taken by researchers. In these approaches, empirical and fundamental methods are preferably used by them. These approaches aim to describe the insulin-glucose dynamics as a couple of mathematical equations that should be easy to manipulate for the insulin therapies and should fully describe the characteristics of the internal insulin-glucose metabolism [13].

Basically, the empirical method uses a model structure (formula or equation) which is determined theoretically with several parameters. The behavior of this model structure is determined by only the input-output data of the system from a number of experiments [13]. In this method, capturing the system behavior or data is the most time-consuming process. In an example of the linear structure of the insulin-glucose system, to represent glucose effects, two parameters are used, and to represent insulin effects, other two parameters are also used in order to close the model to the actual system [13]. In addition to the input-output data, semiempirical method utilizes other physiological factors, such as dynamic behavior and kinetics to create a closer model of diabetic patients [13].

In the fundamental methods, a mathematical representation of the human internal system which is already known sufficiently by researchers constructs an insulin-glucose model. This system behavior includes kinetics and material transport [13]. According to investigating the internal system, a lot of data from the literature can be used to determine the system parameters. Usually the model averages studied behaviors. In particular, in constructing a fundamental diabetes model, the authors in [14] applied the insulin-release data of the *β* cells of the pancreas from a number of examinations to a mathematical representation.

## 4. OPEN-LOOP CONTROL MODELS

Arguably, the most complex component of blood glucose management is the control domain. There are several classes of solutions to this problem, ranging in complexity, prerequisite knowledge, and feedback.

The open-loop system for the insulin-dependent diabetes therapy does not employ any glucose sensors. However, occasionally calling the “open-loop” system is not appropriate and more precisely, the system should be called the “programmed” insulin infusion system because of its incomplete openness. That is, the control loop can be closed by the physician and the diabetic when interacting on the system [15].

One example of the systems is one that was developed by Case-Western Reserve University, and this system is considered to be one of the most intelligent programmed insulin infusion products that deal with the noninsulin-dependent diabetics [15]. The idea is that from an analysis of the insulin curve in the nondiabetic, it was turned out that the curve approximately traces a combination of a double exponential curve and a basal insulin infusion [15]. According to this mathematical model, an intravenous insulin delivery system was designed such that it followed the real pancreas functionality of the nondiabetic. The system utilized a portable cart containing the control system, the insulin-pump, power supplies, and insulin reservoir so that the patient could move around with the devices. The insulin pump delivers low-concentration insulin and updates the insulin delivery rate every 30 seconds. Because of its simplicity, the system can be set up and operated by nurses [15].

This research revealed the diabetics had the blood glucose profile to be improved considerably from a two-week examination, and, moreover, the improved conditions remained even several days after the examination. On the other hand, the researches of this programmed system so far did not indicate any hypocglycemic condition yet [15].

In addition to the achievement of Case-Western Reserve University, Siemens and the Finsen Institute also developed a programmed insulin infusion system that employed a moderately complex delivery algorithm from another approach. The system is capable of manually inserting small pin connectors into the control unit in order to control the insulin delivery rate. Like a product of Case-Western Reserve University, the infusion rate follows an exponential curve, and the insulin infusion rate is updated every 30 minutes [15–18].

## 5. CLOSED-LOOP CONTROL MODELS

**Figure 1:**
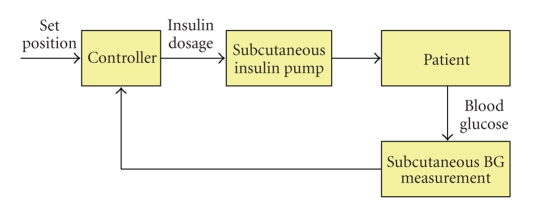
A control flow of closed-loop control models [19]. Entire loop of the control is closed from outside by utilizing an insulin pump and in vivo blood glucose (BG) sensors. Both insulin injections and glucose measurements are carried out subcutaneously.

The system to deliver insulin mechanically in order to regulate the glycemic profile is called the “closed-loop” system [15]. As shown in Figure 1, the closed-loop system completes its operating cycle within the system and no external interaction to diabetic patients is required [19, 20]. In other words, the closed-loop control uses the feedback from the output. Typically, the closed-loop system for type 1 diabetes therapy utilizes the glucose sensor and schematically consists of three phases: blood glucose measurements, insulin demand calculation, and insulin injection. The closed-loop system repeats this sequence. So far, along with the glucose sensor, the closed-loop system also employs an insulin pump which continuously infuses insulin via a subcutaneous root.

Basically, insulin delivery is controlled by these implanted blood glucose sensors and an insulin pump attached to a patient's body [19, 21–23]. In short, according to measurements of glucose level from an implanted blood glucose sensor, an insulin pump continuously infuses insulin into a patient's body. However, although implanted blood glucose sensors benefit a lot for the diabetes therapy, establishing reliable measurements of blood glucose is so difficult that many researches in this field are still under way by many biomedical researchers [19]. Figure 1 shows a control flow of a closed-loop strategy.

Currently many forms of blood glucose sensors exist, such as fingerstick types, implantable types, or noninvasive types. For example, in applying a fingerstick-type blood glucose sensor, blood glucose levels are measured three to seven times a day and according to the measurements, the amount of insulin supply by an insulin pump is updated manually. However, since with the fingerstick-type sensors, measurements are carried out by patients themselves on regular basis, managing patient lifestyle by themselves is rather troublesome. Meanwhile, when using an implantable blood glucose sensor, glucose levels in blood are automatically monitored in a certain amount of period.

In calculating the insulin infusion, many control models are developed by researchers until now: pole-assignment strategy, self-tuning adaptive control, or nonlinear predictive control [19]. More details about these schemes are explained in the later subsections.

### 5.1. Pole-assignment strategy

Pole assignment is a standard control systems technique for designing an infinite impulse response filter [19, 24]. This consists of a set of filter coefficients and a feedback loop in order to maintain a stable blood glucose level.

In general, the closed-loop schemes of the insulin-dependent diabetes therapy utilize an insulin pump that automatically supplies insulin into the human body subcutaneously [19]. Usually the glucose levels are monitored by a needle-type glucose sensor through the subcutaneous (SC) route, and the insulin infusion rate (IIR) is determined by continuous measurements of the blood glucose level. For example, in pole-assignment strategy [21], the IIR in relation to blood glucose level, the insulin-glucose system, is determined by the following computation:

(1) $$\begin{array}{ll} \text{IIR}(t) & =K_{p}G(t)+K_{d}\frac{dG(t)}{dt}+K_{c}\\ \text{with} & \\ K_{p} & =\frac{amnV}{p},\;\frac{K_{d}}{K_{p}}=\frac{1}{l}+\frac{1}{m}+\frac{1}{n}+\frac{b}{a},\\ K_{c} & =d+\frac{c}{a}K_{p}, \end{array}$$

where *G* is blood glucose level and *d* is the insulin infusion rate through the intravenous (IV) route. Parameters *a*, *b*, and *c* can be calculated from the relationship between plasma insulin *I* and blood glucose levels in a normal person, which are written as

(2) $$I(t)=aG(t)+b\frac{dG(t)}{dt}+c.$$

**Figure 2:**
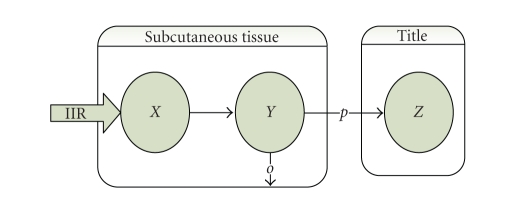
Three-level model of subcutaneous insulin absorption.

Moreover, other parameters *n* and *l* are from next equations which are the pharmacokinetics of insulin infusion through the SC route:

(3) $$\begin{array}{ll} \frac{dX(t)}{dt} & =\text{IIR}(t)+lX(t),\\ \frac{dY(t)}{dt} & =lX(t)-(p+o)Y(t),\\ \frac{dZ(t)}{dt} & =pY(t)-nZ(t),\\ I(t) & =\frac{Z(t)}{V}, \end{array}$$

where *X*, *Y*, and *Z* represent the insulin level in the two subcutaneous compartments and in the plasma, respectively. Figure 2 shows such an *X/Y/Z* 3-level model.

At last, *m* is calculated as *m* = *p* + *o* [19].

This is a simplified approach, forgoing adaptive control for ease of characterization and implementation. For most situations, it will perform as desired, but if it encounters a situation that it handles poorly, it will handle that situation poorly every time it occurs again in the future.

Experiments showed that the combination of the pole-assignment strategy and Lispro insulin generated a similar trend to the use of the IV route with regular insulin [19]. However, the combination of the pole-assignment strategy and regular insulin generated much worse result.

### 5.2. Self-tuning adaptive control

A difficulty of the pole-assignment strategy is to repeatedly evaluate model parameters in each computation of the IIR [19]. To avoid re-evaluations of the model parameters, the self-tuning adaptive control closed-loop scheme employs a recursive assessment of the model parameters so that the glucose level of time period *k*, that is, *G*_*k*_, is evaluated from the glucose levels of time period *k* − 1 through *k* − *h*, that is, {*G*_*k*−1_,…, *G*_*k*−*h*_}, and the insulin doses of time period *k* − 1 through *k* − *p*, {*I* *D*_*k*−1_, *I* *D*_*k*−*p*_}, as well as some unknown parameters Θ, which can be written as [25–28]

(4) $$G_{k}=M\left(G_{k-1},\ldots ,G_{k-h},{ID}_{k-1},{ID}_{k-p},\Theta \right),$$

where *p* and *h* are time delays. Since this method evaluates the blood glucose level of time period *k* from the previous evaluations and insulin doses, it can efficiently eliminate unnecessary re-evaluations of the model parameters.

Besides, according to glucose level *G*_*k*_, the next insulin dose is calculated as

(5) $$J={\left(G_{k}-G_{k-1}\right)}^{2}-r{ID}_{k},$$

such that *I* *D*_*k*_ can minimize value *J*, where *r* is a weighting factor designed to control the amount of insulin dose.

**Figure 3:**
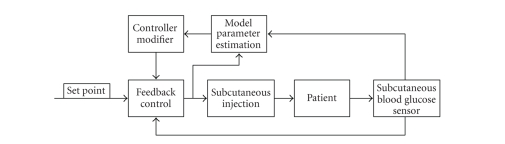
Self-Tuning adaptive control.

Implementation of self-tuning adaptive control, shown in Figure 3, is quite similar to pole-assignment control, as it uses the same system modeling equations in order to compute the insulin infusion rate [19].

The primary difference between the two methods is that another controller is used to constantly evaluate the system model, and may “tune” or redesign the PD controller parameters as needed to obtain more accurate results based upon minimum variance.

One advantage of this control scheme is that it is comparatively easy for a physician to estimate the future trend of blood glucose levels from a set of the past inputs, where the model can be used to predict hypoglycemic or hyperglycemic events before they occur [13].

### 5.3. Model predictive control

**Figure 4:**
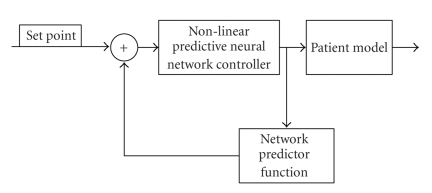
Model predictive closed-loop control.

A model predictive control (MPC), or nonlinear predictive control (NLPC) algorithm attempts to “learn” what nominal means in a system [19, 20], shown in Figure 4. In the case of blood glucose management, a nonlinear MPC algorithm uses sensor data to track glycoregulatory system parameters in order to predict the levels of required insulin infusions. It then uses models of the human glucose metabolism to estimate the effects of the insulin injection. An example of a model used is a nonlinear autoregressive (NARX) model, where previous blood glucose (BG) levels and insulin dosage levels are run through a nonlinear function, often obtained through neural network learning.

Bayesian learning is applied using the model-predicted effect of the insulin, and its actual measured effect. The learning process adjusts system parameters in order to increase the accuracy of its predictions as more iterations are performed. Using this method, the system will become increasingly accurate, and will begin to “understand” how the patient that it is calibrated to will react to insulin injections of varying compositions and strengths.

### 5.4. Nonlinear predictive control (neural predictive control)

**Figure 5:**
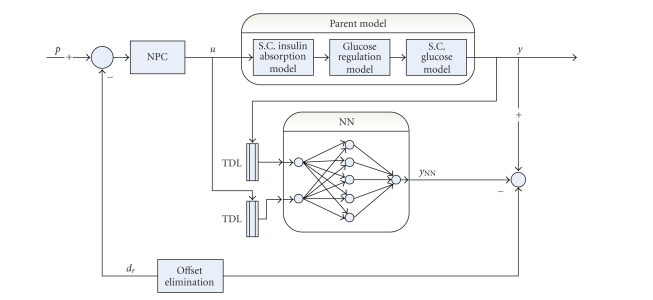
Schematic representation of neural prediction control proposed for the nonlinear predictive control study of the glucose metabolism, which consists of an amalgamation of a neural network and nonlinear predictive control technique.

Apparently, the insulin-glucose system is complicated, and the system is considered to be nonlinear [29]. In [30], in order to follow this nonlinearity, one method utilizes a collaboration of a neural network (NN) and nonlinear model predictive control (NPC) technique, that is, neural predictive control. More precisely, NNs and an NPC are used to simulate the glucoregulatory system. A schematic diagram of the neural predictive control is shown in Figure 5.

Basically NNs approach the problem of blood glucose management without attempting to explicitly describe the exact model of the blood glucose-insulin system [31–33]. This is particularly useful in situations where patients have a disease that complicates normal model description, or an abnormality exists which makes prediction difficult using just measured parameters and sensor data.

Like other control strategies, the main goal of the neural predictive control is to achieve regulation of the glucose profiles for the type 1 diabetics predicting a future glucose curve from the nonlinear model with time delays, so it can follow a similar curve to the metabolism of normal people. A feed forward neural network employing backpropagation can be trained offline using accumulated patient data, including daily blood glucose readings as well as insulin dosages. A neural network will then be able to “learn” based upon experience, much as a human brain learns. This will help it to predict nonlinear behavior, even multiple orders removed, imperceptible to standard data interpretation methods. This capability to be “intuitive” helps to drive a system in which unknowns or immeasurable parameters are still accounted for, and abnormalities are detected and intelligently handled.

The neural predictive control reveals several physiological variables to be controlled. In addition to the control variables, it also designs a cost function in relation to the insulin-glucose model [19, 30].

In the mechanism of the scheme, the neural predictive control makes consecutive control actions toward the glucose metabolism altering the control variables, so that the actions consequently minimize a designed cost function at each sampling time. However, the alteration of the control variables also changes the optimization problem at each sampling time. On the other hand, the model and its parameters can be of no difference during a whole examination [19].

Fortunately, in order to regulate the glucose profile, the so-called monomeric insulin (MI) analogs are currently available. These MI analogs have advantages that they are able to be absorbed through the subcutaneous route three or four times faster than human insulin resulting in that the rise of the plasma insulin concentration grows faster. Besides, the MI analogs are more predicable than human insulin due to its less variability [30].

**Figure 6:**
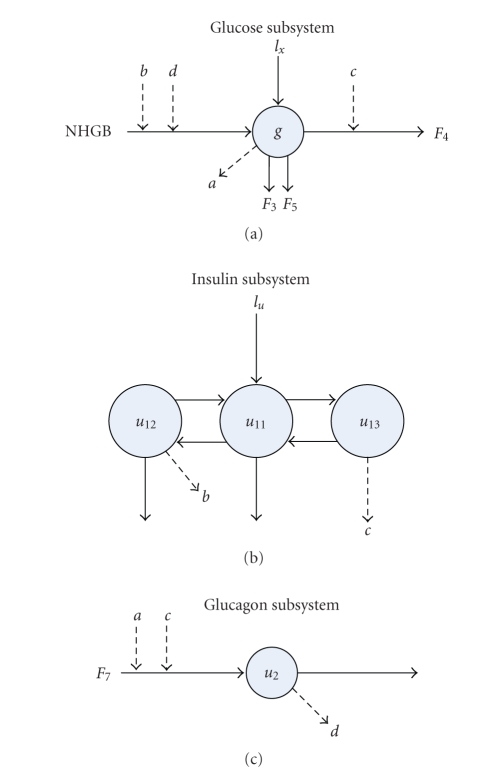
Compartmental models of the glucose regulation system from [30]. Compartment *g* is plasma glucose; compartments *u*_11_, *u*_12_; and *u*_13_ are plasma insulin, liver insulin, and interstitial insulin, respectively, and compartment *u*_2_ is plasma glucagon. External injections of glucose and insulin are represented as *I*_*x*_ and *I*_*u*_. NHGB stands for the net hepatic glucose balance. Nonlinear functions are represented as *F*_*i*_ which depends on variables *a*, *b*, *c*, and *d*.

In the forming of the control strategy, [30] first develops a mathematical model of the insulin-glucose dynamics of the type 1 diabetics, which is mainly broken down into three parts: the subcutaneous insulin absorption model, glucose regulation model, and subcutaneous glucose model, shown in Figure 6. From the model, the subcutaneous insulin absorption is calculated in two steps: the subcutaneous MI analogs infusion and utilization from the subcutaneous depot [34]. With respect to the glucose regulation, in order to model the system mathematically, [30] adopts a compartmental model in which there are single compartments for glucose and glucagon, and three compartments for insulin (liver and portal insulin, plasma insulin, and insulin in the interstitial fluid). Also in the model, net hepatic glucose balance, renal glucose excretion, and glucose utilization are simulated to generate numerical values. From the glucose regulation model, the subcutaneous glucose model is generated by investigating transfer rates between the plasma and subcutaneous compartments. Consequently, the subcutaneous glucose model forms a linear, first-order system with the transfer function [30].

In the second step, using numerical data from simulations of the mathematical patient model, the paper [30] develops the nonlinear system by NNs in order to make future blood glucose predictions. To do this, the paper [30] utilizes a nonlinear autoregressive model (NARX) because of its popularity and usefulness. The form is described as

(6) $$\begin{array}{ll} G_{k} & =f(x_{k})+e_{k}\\ & =f(G_{k-1},\ldots ,G_{k-n_{y}},ID_{k-1},\ldots ,ID_{k-n_{u}})+e_{k}, \end{array}$$

where *G* is a sequence of subcutaneous glucose profiles, especially *G*_*k*_ is a future glucose prediction, *ID* is a sequence of subcutaneous insulin infusion rates, *e*_*k*_ is a noise, and *n*_*y*_ and *n*_*u*_ are both durations of glucose utilization and insulin activation, respectively.

At this point, however, the approximation of the nonlinear function *f* is a hard task. A reason why to approximate the nonlinear function is difficult is that the function is required to be made up from finite data although there are usually infinite solutions for it. To resolve this difficulty, approximation based on regularization techniques is used because it was proved that regularization principles consequently can derive networks with one layer of hidden units, that is, regularization networks [30]. Thus, the paper [30] uses a function of radial basis function (RBF) networks, which are a subclass of regularization networks, in order to represent the nonlinear function *f* :

(7) $$\begin{array}{cc} f(x(t)) & =\overset{n}{\underset{i=1}{\sum }}w_{i}H(∥x-x_{i}^{0}∥),\\ H(∥x-x_{i}^{0}∥) & =\frac{1}{{\left({\left(x-x_{i}^{0}\right)}^{2}+\beta \right)}^{2}}, \end{array}$$

where *H* is a continuous function of **R** ^+^→**R**, || ||, represents the Euclidean norm, *x*_*i*_^0^ are some proper center values selected from the data points, *w*_*i*_ are some weight constants, and *β* is a parameter representing the dispersion [19, 30]. Also the regularized orthogonal least squares (ROLSs) algorithm is used to determine RBF weights and centers in order for *f*(*x*(*t*)) to fit the data under some constraints [19].

The third step to model the control scheme is to design an NPC [30]. To do this, the moving horizon approach is used to obtain the control law. Thus, eventually controlling the glucose profiles is turned into resolving the minimization problem where a sequence of subcutaneous insulin infusions must sufficiently minimize the problem below

(8) $$\arg\underset{ID}{\min}\;J=\left[\overset{N_{p}}{\underset{j=N_{1}}{\sum }}(e_{j}^{T}\Gamma e_{j})+\overset{N_{c}-1}{\underset{j=0}{\sum }}\Delta ID^{T}(t+j)\Gamma_{u}\Delta ID^{T}(t+j)\right],$$

where *I* *D* = [*I* *D*_*k*_, *I* *D*_*k*−1_,…, *I* *D*_*k*−*N*_*c*_−1_]^*T*^ and *N*_*c*_, *N*_*p*_, *N*_1_, Γ_*e*_, Γ_*u*_ are parameters of the controller for tuning.

### 5.5. Fuzzy control scheme

A fuzzy control scheme is studied in [35]. There are three steps for the process of a fuzzy logic algorithm: fuzzification, rules, and defuzzification.

1. Fuzzification: the input of a controller is an exact number, like the concentration of glucose is 100 mg/dl. What the fuzzification do is to fuzzy the concentration such as low concentration, high concentration, and proper concentration. Every exact number has the weight of all these low concentration, high concentration, and proper concentration.
2. Rules: after defining the fuzzy concept of input, rules are made to decide what the output should be: more drug, a little drug, or no drug.
3. Defuzzification: after the rule, the output of fuzzy concept is obtained, for example, more of 0.8 and little of 0.2. But the output which is the object model's input must be an exact number, that needs to be defuzzification. By defuzzification, the output gets an exact number.

In the paper [35], it is assumed that there are two different inputs of the concentration of glucose and the change rate of concentration, and one output of the dose of drug. “overlow,” “good,” “high,” and “overhigh” are defined for the concentration. The rate is “overlow,” “low,” “high,” and “highest.” The dose of drug is defined as “zero,” “little,” “norm,” “more,” and “most.” Ten rules are defined such that [35]:

1. if (rate is overlow), then (dosage is zero);
2. if (concentration is overhigh) and (rate is low), then (dosage is little);
3. if (concentration is overhigh) and (rate is highest), then (dosage is most);
4. and so forth.

## 6. PARTIAL CLOSED-LOOP SCHEME

**Figure 7:**
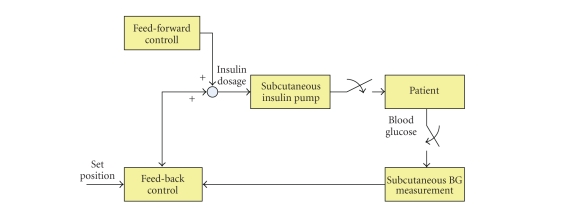
A control flow of partially closed-loop insulin therapy models from [19]. In general, in this case, insulin dosages are supported by an expert system that generates an optimal insulin dosage from a comparison between a model case and a prediction of a future blood glucose transition.

In a partially closed-loop scheme of the insulin-dependent diabetes therapy, measurements are conducted three to seven times per day, and insulin injections are also performed three to four times under the supervision of a physician. These decisions, for example, the number and type of insulin injections, insulin dosage [19], are made according to model-based or algorithmic-based decision support systems, such as DIAS, AIDA, and T-IDDM [19]. Insulin injections are usually performed by using the subcutaneous (SC) route due to its management and safety. Also there is an alternative route for insulin delivery that is ideal for control, the intravenous (IV) route. However, this route is not ideal for the management and safety. Figure 7 shows a control flow of a partial closed-loop scheme.

While in the closed-loop systems, the blood glucose levels are automatically monitored by an implantable sensor and according to the measurements, insulin infusions are carried out in the use of an insulin pump or three or four times of the insulin injections, in the partial closed-loop, the metabolic controls partly rely on the physician's evaluations of the measurements of the blood glucose levels, the amount of insulin injections and physical exercises as well as glycosuria and ketonuria [19]. Moreover, as other metrics of the evaluation, medium period indicators, such as glycated hemoglobin (HbA1c), are captured, where the blood glucose levels of the past 60 days can be seen. These data are recorded by the patients everyday. In other words, the partial closed-loop scheme is made out of a collaboration of the feedforward and feedback controls, and usually feedforward controls are made by clinicians who determine it from the patient's lifestyles. However, these evaluations largely rely on doctor's experiences.

From the objective of the insulin therapy that aims to reconstruct the artificial insulin metabolisms in relation to the levels of blood glucose in the body, it is typically an optimization problem that is viewed from four dimensions: the number of injections, time of injection, insulin type, and insulin dosage [19]. Basically, this four-dimensional space optimization is intrinsic.

Besides, occasionally a clinician provides him/her with a feedforward strategy from the patient's lifestyle.

### 6.1. Physician prescribed regiment

**Figure 8:**
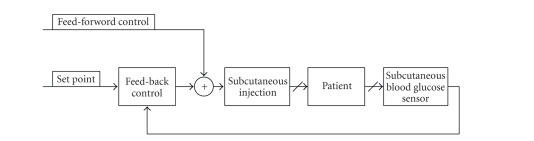
Partially closed-loop control.

The insulin regiment prescribed by a doctor, to be administered manually, constitutes partially closed-loop control [19, 24]. A physician will dictate an insulin administration routine to a patient, variant upon a patient's lifestyle. Patients under such a system monitor their blood glucose level several times a day, administering insulin based upon prescribed tables according to their schedule and BG level. Figure 8 shows the procedure.

This is the method that has traditionally been used by insulin-dependent diabetics, but it performs poorly compared to other methods. Partially, closed-loop control is far from real time and only updates its control routine at scheduled physician visits. Furthermore, lifestyle events such as eating, sleeping, or working out must be accounted for by the patient in their interpretation of insulin tables, introducing the very real danger of human error.

### 6.2. The Bergman model

The Bergman model is one of the virtual diabetes patient models represented in the literature. In the Bergman model, the certain dynamics of the diabetes patient system can be represented as mathematical equations by employing three-order model: a glucose compartment, *G*, a remote insulin compartment, *X*, and an insulin compartment, *I* [4, 36, 37]. Basically, the Bergman model has a very simple form and is represented as follows:

(9) $$\begin{array}{ll} \frac{dG(t)}{dt} & =-(p_{1}+X(t))G(t)+p_{1}G_{b},\\ \frac{dX(t)}{dt} & =-p_{2}X(t)+p_{3}(I(t)-I_{b}),\\ \frac{dI(t)}{dt} & =\gamma (G(t)-h)t-nI(t), \end{array}$$

where *G*(*t*) represents plasma glucose and *I*(*t*) represents plasma insulin at certain time *t*, which are initialized at *t* = 0. *X*(*t*) represents the effect of insulin causing net glucose disappearance, for example, the remote insulin concentration. *G*_*b*_ represents a base value of plasma glucose and likewise, *I*_*b*_ represents a base value of plasma insulin.

Moreover, *p*_1_ is a parameter representing glucose effectiveness, *p*_2_ fractionally represents the insulin-dependent increase rates and *p*_3_ fractionally represents the net remote insulin disappearance rates. *h* is a threshold where the plasma glucose levels are expected not to exceed. When the plasma glucose levels exceed the threshold, the second-phase of insulin secretion will be performed with additional *γ* insulin secretion. Furthermore, insulin is removed from the plasma insulin space at rate of *n*.

The Bergman model is used for efficiently predicting the certain diabetes patient system dynamics.

### 6.3. Automated insulin dosage advisor

The automated insulin dosage advisor (AIDA) is a virtual diabetes patient model that was originally designed for the educational purpose so as to help patients and clinicians learn effective glycemic control [4, 38]. Basically, AIDA is used for estimating effects of insulin injections and regimens in type 1 diabetic therapy [39]. More precisely, AIDA is a simulation program that models the insulin-glucose dynamics based on the physiological rules around metabolism of a single glucose compartment [19, 38].

**Figure 9:**
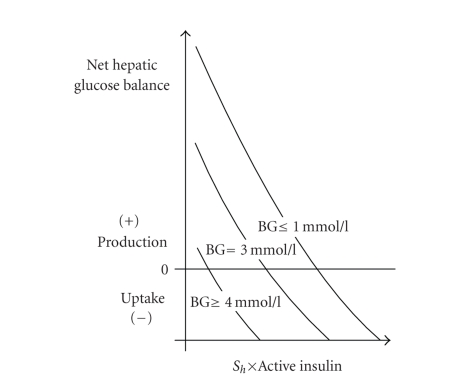
Variation of the net hepatic glucose balance in terms of the changes of the amount of the active insulin and blood glucose (BG) levels from [39]. The figure demonstrates that the amount of the active insulin reduces the net hepatic glucose balance. The transitions of the net hepatic glucose balance depend on a liver sensitivity parameter *S*_*h*_. A value of *S*_*h*_ is within the range of 0 to 1.

To model the insulin-glucose dynamics, AIDA prepares a single glucose pool (compartment) of extracellular glucose. Metabolism around the glucose compartment is carried out such that delivering glucose into the compartment is conducted both by being absorbed by the intestine and by being produced by the liver (gluconeogenesis). On the other hand, removing glucose out of the compartment is carried out by insulin-independent and insulin-dependent glucose utilization. More precisely, by the insulin-independent glucose utilization, glucose is carried from the compartment into red blood cells, the central nervous system and viscera, whereas by the insulin-dependent glucose utilization, glucose is carried from the compartment into the liver and periphery. To hepatic and peripheral glucose utilization, AIDA is designed to have a capability of adding different insulin sensitivities by modeling them separately. Besides, the renal threshold is defined in order to model renal glucose losses [38, 39]. Figure 9 shows a variation of the net hepatic glucose balance in terms of the changes of the amount of the active insulin and blood glucose (BG) levels in the form of nomograms.

Moreover, the AIDA model applies a process of insulin absorption derived by Berger and Rodbard to its pharmacokinetics of the model [40].

In addition, insulin is separated into two compartments where one is for plasma insulin and the other is “active” insulin. On the basis of a physiological model, “active” insulin controls metabolism. On the other hand, hepatic degradation produces insulin from plasma insulin [39].

In the use of AIDA, by comparing home-monitored blood glucose levels to a typical behavior of blood glucose of a patient, for example, the model day, metabolic problems are identified. According to particular metabolic problems, AIDA can generate several possible solutions, and among these possible solutions, one best solution will be selected according to a nonlinear dynamic model of the insulin-glucose system.

At last, this nonlinear dynamic model of AIDA consists of four differentials, 11 algebraic equations and 17 parameters [41]. From the data, the model will be constructed while simulations are performed for each therapeutic choice to optimize the following cost function:

(10) $$J(G)=\int_{0}^{T}{\left(G(t)-G_{0}\right)}^{2}dt,$$

where *G*_0_ describes a set point [19].

### 6.4. Diabetes advisory system

The diabetes advisory system (DIAS) is a nonlinear model of the blood glucose-insulin system based upon real-life parameters, versus simply BG measurements [21]. It incorporates qualitative and quantitative input from the user, including BG levels, meals, and past insulin injections.

**Figure 10:**
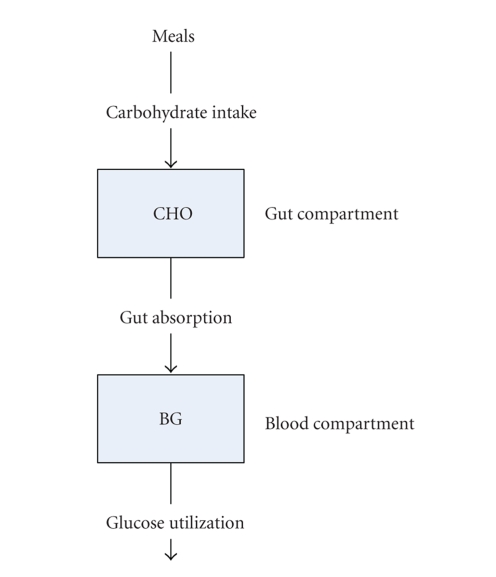
The flow of the physiological calculation of carbohydrate by the diabetes advisory system (DIAS) from [42]. There are two state variables in the model. CHO stands for the amount of carbohydrate in the gut compartment and BG stands for that in the blood compartment. In the figure, the carbohydrate intake raises the level of CHO and likewise, the gut absorption raises the level of BG. On the other hand, the glucose utilization lowers the level of BG.

The system uses a discrete-time finite-state model of the system based upon user input. The system uses what it understands about the system as a whole, including dormant compartmentalized insulin, predigested carbohydrates, and current BG levels to compute a Bayesian estimate of future BG levels. It uses all known information in order to compute the value of the optimal dosage such that it minimizes an associated cost function (i.e., hypoglycemia is far more “costly” than hyperglycemia, due to its possibility of severe damage). Over iterations, it will adjust its system model parameters to better account for patient specific reactions it detects. For example, in DIAS, the flow of the physiological calculation of carbohydrate is drawn by Figure 10 [42]. Besides, DIAS has three different modes, for example, the learning mode, the prediction mode, and the advisory mode.

In Figure 10, there are two associated state variables, for example, CHO and BG, and both of them record how much carbohydrate content exists in the gut compartment and the blood compartment, respectively [42, 43]. For example, in this model, carbohydrate content is, at first, taken in from meals and delivered to the gut and the blood compartment. During the delivery to the blood, some of carbohydrate content is absorbed by the gut and only the rest of it is delivered to the blood and transformed into the main energy.

**Figure 11:**
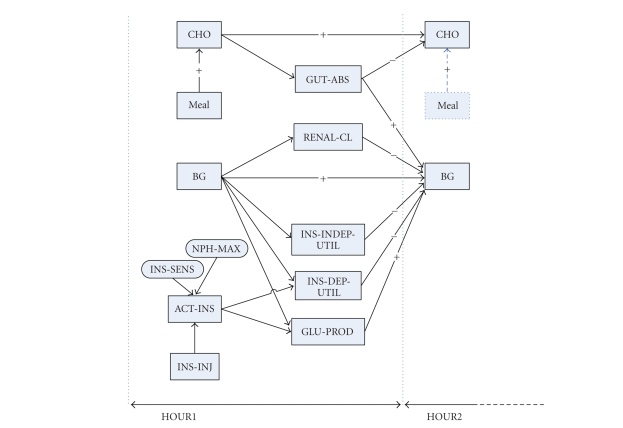
Another form of the insulin-glucose dynamics which is described by difference equations from [42]. This figure shows that the amounts of carbohydrate in both the gut and blood are calculated every hour. Basically, these calculations are made depending upon absorptions and utilization. For example, the figure shows the difference equations between hour 0 and hour 1.

In addition, this flow of carbohydrate is redrawn more precisely by using difference equations, which are shown in Figure 11. In this redrawn model, the amount of carbohydrate content in both the gut (CHO) and blood (BG) compartment is updated in every hour such that both increase and decrease of carbohydrate content are kept track of in each compartment. In Figure 11, the GUT-ABS process variable represents how much glucose is absorbed by the gut, and the rest of glucose remains in the gut and is recorded in the CHO state variable. Besides, the RENAL-CL process variable represents how much glucose is removed, the INS-INDEP-UTIL process variable represents how much glucose is used independently of insulin, the INS-DEP-UTIL, on the other hand, represents the amount of glucose to be used for insulin-glucose dynamics, and the GLU-PROD process variable represents the amount of glucose produced by the liver [42, 43].

In DIAS, there are also two input variables, for example, MEAL and INS-INJ, where the MEAL input variable is carbohydrate intakes at given time from each meal, and the INS-INJ input variable represents the amount of the external insulin injection [42, 43].

Furthermore, to adjust individual physiological differences, two other parameters are employed, for example, INS-SENS and NPH-MAX, where the INS-SENS parameter stands for the insulin sensitivity that affects the active insulin (ACT-INS) variable, and the NPH-MAX parameter represents the time when NPH insulin achieves the maximum absorption or concentration, shown in Figure 11 [42, 43].

The “+” and “−” symbols in Figure 11 represent fluctuation of carbohydrate on the CHO and BG state variables. For example, the CHO state variable at HOUR 1 can be written as an equation such that CHO (HOUR 1) = CHO (HOUR 0) – GUT-ABS (HOUR 0) + MEAL (HOUR 1) [42, 43].

The transition among the states in a graph of difference equations in DIAS is defined by causal probabilities. More precisely, the graph representation of difference equations of DIAS is defined by a causal probabilistic network (CPN) or a Bayesian network using the HUGIN approach [42, 43]. For example, the transition probability of the amount of carbohydrate absorbed by the gut given the carbohydrate content in the gastrointestinal tract is P(GUT-ABS ∣ CHO) [42].

As previously mentioned, there are three modes in DIAS to conduct a calculation of the optimal insulin dosage (the decision support system): the learning mode, the prediction mode, and the advisory mode.

At first, the learning mode is used to generate the two adjustable parameters, for example, the insulin sensitivity (INS-SENS) and time-to-peak absorption of NPH (NPH), from the collection of standard data, such as the amount of blood glucose, insulin injection, and carbohydrate content in the meals [42, 43]. For instance, an example of a prediction of blood glucose transition from [42], from the measurements of blood glucose, the mixture of short-acting insulin and intermediate-acting insulin and the carbohydrate intake, the transition of blood glucose is predicted as a straight line.

Secondly, the objective of the prediction mode is to predict the resulting blood glucose concentration from given an intake of carbohydrate and insulin injection as well as two adjustable parameters estimated in the learning mode [42, 43]. There is a risk of hypoglycemia around lunch time, the ratio of the insulin mixture is manually changed in the morning to handle the condition. As an example of the objective of the prediction mode of DIAS given from [42], there is a risk of hypoglycemia around lunch time, the ratio of the insulin mixture is manually changed in the morning to handle the condition. In DIAS, this mode calculates an effect of a manually modified insulin therapy.

At last, the advisory mode, which is considered as a special version of the prediction mode, is used to generate possible insulin therapies that avoid the overall risk of an excess or shortage of blood glucose by minimizing an utility measure. In the advisory mode, the manual changes of the insulin regimen conducted in the prediction mode, such as switching to a different mixture of insulin, are automatically replaced to an optimal way by the system. For example, a replaced version of an optimal insulin injection procedure is used by the advisory mode, where the mode recommends reducing the amount of NPH insulin from 10 to 6 U before dinner resulting in avoiding hypoglycemia during bedtime [42, 43]. As an example of a result calculated by the DIAS advisory mode, DIAS further generates an optimal solution of an insulin therapy automatically to avoid the overall risk of an excess or shortage of blood glucose by minimizing a utility measure.

### 6.5. Telematic management of insulin-dependent diabetes mellitus

**Figure 12:**
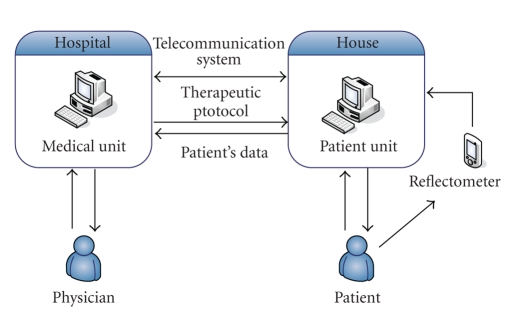
The T-IDDM project architecture from [44].

The EU developed telematic management of insulin dependent diabates mellitus (T-IDDM) which was a telemedicine system that supported clinician's decision-making for providing insulin for the insulin-dependent diabetics. Basically the system consist of two modules, for example, a patient unit (PU) and a medical unit (MU), and two decision support elements, for example, a rule-based reasoner (RBR) and a case-based retrieval system (CBRS) [19, 44, 45]. The system architecture is shown in Figure 12.

With the system, the PU is basically responsible for monitoring changes of the blood glucose concentration in patients, and the physiological data are transferred to the hospital database via the Internet. Both manual and automatic measurements and data transfers are allowed. In addition to the hospital database, the PU also has a local database that enables patients to deal with their own diabetics cases autonomously [44].

On the other hand, the MU is responsible for supporting the clinician's sides of functionality. Thus the MU can visualize incoming data from patients, analyze them, and generate optimal insulin treatments for particular patients. Basically, the MU is a web-based application made up of collaborating five servers: a database server, temporal abstraction server, data analysis server, decision support system, and web server [44]. Data communication is made up between the two devices.

From the perspective of the decision support for the therapy, the RBR and CBRS are designed to resolve the following premises.

At first, the insulin-glucose dynamics is very complicated that only highly parameterized nonlinear models can be applied to it although it is usual to have no more than three or four blood glucose tests per day in the insulin-dependent diabetes therapy, which is not enough to model the dynamics. Due to the limitation of the measurements, some parameters are required to be fixed in all cases whereas only a few parameters are free to be set up according to each patient case. However, this limitation of setting parameters must make the models and the quality of prediction inflexible and inaccurate [19]. One goal of T-IDDM is to refine the models to generate more precise prediction of the insulin-glucose models from the limited inputs.

Secondly, the insulin therapy largely usually depends on experiences of the professionals. T-IDDM provides the professionals with more systematical way to plan the therapy for particular insulin-dependent diabetics.

At last, the same metabolic behavior occasionally generates different results. For example, either “honeymoon” effect or other troubles may cause the same number of hypoglycemia over a month. However, this type of wrong diagnosis may fall the diabetic into a critical situation. Therefore, in independence of metabolic behaviors, T-IDDM should employ context that can generate different results from the same metabolic behavior. Also the introduction of context may reduce the search space from whole possible solutions [19].

In the implementation of the RBR, to optimize the therapy, it runs four sequential tasks each of which is connected to a set of rules through a forward chaining mechanism. These four sequential tasks are the data analysis, problem identification, suggestion selection, and therapy revision [44].

However, only the RBR is sometimes not enough to produce reliable suggestions of the therapy for poorly controlled patients. Therefore, in consideration of that situation, in addition to the RBR, T-IDDM employs the CBRS to improve the system to be more accurate. The objective of the CBRS is to search a pool of past cases for similar situations to the current condition and utilize them to help the user make an optimal decision for the current condition [44]. This case retrieval follows two steps. In the classification step, a set of past cases is narrowed for searching according to very high-level view of the cases by a Naïve Bayes strategy [46], and after that, in the proper retrieval step, cases having the closest to the current situation are effectively chosen and shown to the user [19].

### 6.6. Insulin-glucose system

Regarding the artificial insulin-glucose control system, [47] designs a fuzzy logic reasoning system. There are mainly two modules (e.g., an analog signal conditioning board and a microcontroller board) and other interface devices, such as a LCD display, a sixteen button keypad, and alarm system, along with the operating software to work the system. The software operates the system according to a fuzzy logic reasoning method. In the system, an analog signal conditioning board is responsible for generating electrical signals from vital parameters monitored by several biomedical sensors. These parameters include sweating, snoring, heart rate, and EEG. In the meantime, a microcontroller board processes these electrical signals. The system works with batteries so that it can be portable.

In the use of the system, several electrodes are placed on the particular segment of the human body capturing the four physiological parameters. Basically, these electrodes are connected to an analog signal conditioning board that amplifies and filters the vital signs. Since a 10 bit A/D converter interfaces a microcontroller board, the analog signals can be converted to the digital signals with the sampling rate of 100 samples/s. The microcontroller invokes the fuzzy logic reasoning algorithm that resides on the ROM of the system to process the four parameters.

## 7. CONCLUSION

This survey mainly focuses discussions on control methods for the insulin-dependent diabetes (type 1 diabetes). Three control methods are introduced in this paper, namely, open-loop, fully, and partially closed-loop control methods. In either of them, the objective of the control methods is to suppress the blood glucose profiles to avoid a condition of hyperglycemia. Because diabetes is a metabolic disorder which is characterized as complete or partial lack of insulin functionality, therapies can be done by making up for the lack of insulin supply by exogenous insulin replacement.

In the open-loop control method, the insulin replacement is programmed such that the amount of the insulin supply follows the non-diabetic insulin delivery. The open-loop control method usually does not count on utilization of blood glucose sensors, but instead, a transition of the insulin supply is captured by carefully examining the nondiabetic in advance.

On the other hand, fully and partially closed-loop controls typically rely on feedbacks from the blood glucose sensor measurement. In the closed-loop control method, the system loop is fully closed so that it does not require any assessment by physicians. It is only based on feedbacks from one or more blood glucose sensors, and it usually requires continuous glucose measurements. Thus from the measurements of blood glucose profiles, a rate of the insulin supply by an insulin pump is adjusted so that it can lead to neither conditions of hyperglycemia nor hypoglycemia. Examples of the closed-loop control method are pole-assignment model, self-tuning adaptive control, model predictive control, and nonlinear predictive control.

In addition to the blood glucose sensor feedbacks, partially closed-loop control also relies on feedforwards by physicians. In the partially closed-loop control method, the blood glucose measurements are conducted three to seven times per day and insulin injections are done three or four times per day. Although both the blood glucose samples and insulin injections are discrete, these are compensated by physicians’ feedford assessment of insulin requirements. Usually a calculation of the insulin supply utilizes a flow chart or table which describes complex relations between blood glucose reduction and insulin supply. Examples of the partially closed-loop control method includes Bergman model, automated insulin dosage advisor (AIDA) and diabetes advisory system (DIAS).

Currently, diabetes is considered incurable. Hence challenges of the blood glucose control methods are to delay the emergence of diabetic complications but not cure the patients from diabetes. In practice, three control methods introduced in this survey partially satisfy these requirements. However, our expectation is that the progress of the technology will enable the construction an “artificial pancreas” that follow the regular functionality of the actual pancreas, and this new technology must lie on the current control technologies.
